# Supplementary material for: Post-stroke health-related quality of life following lower-extremity constraint-induced movement therapy - An observational survey study
Source: PLoS One. 2025 May 8;20(5):e0323290. doi: 10.1371/journal.pone.0323290 (PMC12061391; doi:10.1371/journal.pone.0323290)
Supplement: Attachment — Submitted filename: LE-CIMT.docx [file pone.0323290.s001.docx]

This study uses the Rand-36 questionnaire to evaluate QOL for individuals who had LE-CIMT therapy after stroke and then compares it to data from the general population. The authors failed to convince me why they are comparing healthy individuals to stroke patients instead of stroke patients who received LE-CIMT to those who received conventional therapy. I think that this would be a more interesting comparison to see if LE-CIMT helps improve QOL.

I would need stronger rationale for the study they did, other than that the normative data for healthy individuals was readily available. Having said that, the methods are sound and publishable if stronger rationale for the study is provided.

Abstract

1. Why healthy population as opposed to people who had stroke but did not have LE-CIMT?
2. Line 29-30. 90% could move inside and outside, but 21% were inactive. I would clarify as this is over 100%. I assume that some participants who could get around still considered themselves inactive?
3. I guess I am not surprised by the results. Someone who had a stroke is going to score less on QOL than a healthy control. I think the real question is if you had a stroke and LE-CIMT, was there differences for people who had a stroke and didn’t receive LE-CIMT.

Introduction

1. Seems like Zhou et al compared LE-CIMT to conventional therapy and showed promising results. I would have confirmed this information in your data set.
2. Line 75-79. You state that we need to compare healthy controls to stroke who had LE-CIMT. Why? Why would this provide useful individualized rehabilitation interventions. You need to really increase your rationale here. Either, switch to include conventionally treated stroke participants, or convince me this is warranted to study.

Methods

1. Why were participants excluded if they had <3 hours of LE-CIMT. They were included if they had 6 hours LE-CIMT. What happens if they had 4.5 hours LE-CIMT?

Results:

1. Line 164: fix weremale
2. Table 1: how was activity levels defined. IE, what was some light physical activity? Were examples of light activity given? What about regular physical activity?
3. There are no error bars on figure 2. These should be included
4. Table 2. Why was sex used to differentiate different responses for Rand-36? Was there a rationale for this? Why not age? Race? Or degree of stroke? Etc.

Discussion

1. Lines 242-245. The different scores cannot be extrapolated to mean there was long term positive effects of LE-CIMT. This should be removed.
2. Lines 254-260. Once again, I don’t think you can draw this conclusion from these results. The higher scores may reflect less severe initial strokes, or other factors other than the utility of LE-CIMT. You would need a randomized trial comparing LE-CIMT to conventional therapy to make this clain.
