# Supplementary material for: Post-stroke health-related quality of life following lower-extremity constraint-induced movement therapy - An observational survey study
Source: PLoS One. 2025 May 8;20(5):e0323290. doi: 10.1371/journal.pone.0323290 (PMC12061391; doi:10.1371/journal.pone.0323290)
Supplement: Attachment — Submitted filename: Response to Reviewers.docx [file pone.0323290.s003.docx]

# Response to Reviewers

Reviewer #1: The manuscript is well detailed and the methods used is explicit . it is written in standard English as well. Thanks
can the topic be revised to "A cross-sectional evaluation of post-stroke health-related quality of life following lower-extremity 4 constraint-induced mobility therapy" The topic is now revised.

Reviewer #2: Please add MeSH related keywords. In the PLOS one manuscript body formatting guidelines there is no information about MESH-related keywords at all. If it is ok with keyword I would like to add them.
"who had experienced a stroke and had previously participated in LE-CIMT" The methodology is not clear. Action taken. The sentence is clarified.
Why do you name the study as cross-sectional? Thank you for bringing this to our attention. The topic is now revised and also the method section.

pre and post-procedure results should have been compared. I agree, but it was not possible under my time as a PhD-student. I started my PhD-period in 2020 and the intervention with LE-CIMT ended 2018. I had only possibilities to collect post- intervention data of RAND-36.
Some data were collected retrospectively. Yes, their previously results on 6MWT were collected retrospectively. The sentence is now clarified in the method section.

Reviewer #3: The lack of pre-intervention HRQoL data prevents direct comparisons to baseline levels. Yes I agree, there is a lack and in forthcoming studies pre-intervention data should be included.
Potential selection bias due to non-respondents , should be discussed further. Action taken. I have now further developed the discussion around selection bias and non-responders

Explore additional interventions that complement LE-CIMT, such as cognitive and psychosocial support. Action taken. I have now further developed the discussion about additional interventions.
Include qualitative assessments to better understand the lived experiences of post-stroke patients undergoing LE-CIMT. I agree, and a qualitative study were performed earlier. ”I got knowledge of myself and my prospects for leading an easier life”: Stroke patients’ experience of training with lower-limb CIMT. <https://www.tandfonline.com/doi/full/10.3109/14038190903141048>
Highlight specific recommendations for integrating LE-CIMT into post-stroke rehabilitation protocols. Action taken. The discussion section has been revised.
Emphasise the need for longitudinal studies to confirm the long-term impact of LE-CIMT. Action taken. The discussion section has been revised.

Reviewer #4: Please see the attach for specific comments. These comments should be shared with the author. See table below, I have answered all the questions in the attached file
I responded partly for question 1 as some of their conclusions are overstated in the discussion given that this is an observational survey study. Action taken. The discussion section has been revised.

| **Reviewer’s questions:** | **Action taken and Comments:** |
| --- | --- |
| Abstract |  |
| 1. Why healthy population as opposed to people who had stroke but did not have LE-CIMT? | Action taken, the rationale is clarified. |
| 2. Line 29-30. 90% could move inside and outside, but 21% were inactive. I would clarify as this is over 100%. I assume that some participants who could get around still considered themselves inactive? | Action taken, the sentence is now clarified. |
| 3. I guess I am not surprised by the results. Someone who had a stroke is going to score less on QOL than a healthy control. I think the real question is if you had a stroke and LE-CIMT, was there differences for people who had a stroke and didn’t receive LE-CIMT. | Yes, there is a need of more RCT to confirm the effects of LE-CIMT (6 hours a day for 2 weeks) compared with conventional therapy.  I was surprised that there was no difference when it comes to pain and energy/fatigue. |
| Introduction |  |
| 1. Seems like Zhou et al compared LE-CIMT to conventional therapy and showed promising results. I would have confirmed this information in your data set. | I don't understand what you want me to revise, can you clarify your question, please |
| 2. Line 75-79. You state that we need to compare healthy controls to stroke who had LE-CIMT. Why? Why would this provide useful individualized rehabilitation interventions. You need to really increase your rationale here. Either, switch to include conventionally treated stroke participants, or convince me this is warranted to study. | Action taken  The sentence is revised to improve the rationale for the study . |
| Methods |  |
| 1. Why were participants excluded if they had <3 hours of LE-CIMT. They were included if they had 6 hours LE-CIMT. What happens if they had 4.5 hours LE-CIMT? | Yes, that’s right we only included those who had completed LE-CIMT 6h/day, because we reused their result (6MWT) from our previous study and the inclusion criteria for that study were LE-CIMT 6h/day. |
| Results: |  |
| 1. Line 164: fix weremale | Thanks now it's fixed |
| 2. Table 1: how was activity levels defined. IE, what was some light physical activity? Were examples of light activity given? What about regular physical activity? | We used the Saltin-Grimby Physical Activity Level Scale (SGPALS) with this definition of light and regular physical activity.  **Some light physical activity**: Physically active for at least 4 hours/week, such as riding a bicycle or walking to work, walking with the family, gardening, fishing, table tennis, bowling etc.  **Regular physical activity and training**: Spending time doing heavy gardening, running, swimming, playing tennis, badminton, calisthenics and similar activities, for at least 2-3 hours/week  The whole version of SGPALS is available here  chrome-extension://efaidnbmnnnibpcajpcglclefindmkaj/https://mellanarkiv-offentlig.vgregion.se/alfresco/s/archive/stream/public/v1/source/available/sofia/hos1697-550018648-18872/native/SGPALSeng.pdf |
| 3. There are no error bars on figure 2. These should be included | Action taken, error bars included in figure 2. |
| 4. Table 2. Why was sex used to differentiate different responses for Rand-36? Was there a rationale for this? Why not age? Race? Or degree of stroke? Etc. | We only had data, disaggregated by sex, from the norm-based data set for comparison. |
| Discussion |  |
| 1. Lines 242-245. The different scores cannot be extrapolated to mean there was long term positive effects of LE-CIMT. This should be removed. | Action taken  The sentence is revised. |
| 2. Lines 254-260. Once again, I don’t think you can draw this conclusion from these results. The higher scores may reflect less severe initial strokes, or other factors other than the utility of LE-CIMT. You would need a randomized trial comparing LE-CIMT to conventional therapy to make this clain. | Action taken  The sentence is revised. |
