# Supplementary material for: Post-stroke health-related quality of life following lower-extremity constraint-induced movement therapy - An observational survey study
Source: PLoS One. 2025 May 8;20(5):e0323290. doi: 10.1371/journal.pone.0323290 (PMC12061391; doi:10.1371/journal.pone.0323290)
Supplement: Attachment — Submitted filename: Response to Reviewers2.docx [file pone.0323290.s004.docx]

# Response to Reviewers

Journal Requirements:

Please review your reference list to ensure that it is complete and correct. If you have cited papers that have been retracted, please include the rationale for doing so in the manuscript text, or remove these references and replace them with relevant current references. Any changes to the reference list should be mentioned in the rebuttal letter that accompanies your revised manuscript. If you need to cite a retracted article, indicate the article’s retracted status in the References list and also include a citation and full reference for the retraction notice.

Thanks for bringing this to our attention. I have gone through the reference list and where the DOI has been missing I have inserted the PMID instead.

One reference Acarös Candan S, Livanelioglu A. Efficacy of modified constraint-induced movement therapy for lower extremity in patients with stroke: Strength and quality of life outcomes. Turk J Physiother Rehabil 2019;30(1):23-32. <https://doi.org/10.21653/tfrd.406349>. Has been replaced, I can´t find that the article has been retracted but the article is not searchable with PubMed so I chose to replace it anyway.

Reviewer #1: (No Response)

Reviewer #2: Re-review of the manuscript: Post-stroke health-related quality of life following lower-extremity constraint-induced movement therapy - An observational survey study
Comments:
Some of the queries are not addressed properly. I am sorry, can you clarify which queries who are not addressed properly?

Reviewer #3: The manuscript is well-structured and contributes valuable knowledge to post-stroke rehabilitation. Minor refinements in the discussion and methodological transparency would further strengthen its impact. I recommend acceptance with minor revisions.
Suggestion: 1. Consider discussing how LE-CIMT compares to other gait rehabilitation strategies in improving HRQoL. Thanks, action taken. I have now further developed the discussion around how LE-CIMT improving HRQoL compares to other gait rehabilitations strategies
2. Discussing why some HRQoL domains were not significantly different from the general population could enhance interpretation. Thanks, action taken. I have now further developed the discussion around the domains who were not significantly different from the general population.

Reviewer #4: Thank you for the corrections. I did notice some typos in the new manuscript, in particular the discussion which should be corrected. Thanks, I hope I found and correct all the typos you had notice
